# Self-Evaluation of PANDA-FBG Based Sensing System for Dynamic Distributed Strain and Temperature Measurement

**DOI:** 10.3390/s17102319

**Published:** 2017-10-12

**Authors:** Mengshi Zhu, Hideaki Murayama, Daichi Wada

**Affiliations:** 1 Graduate School of Engineering, The University of Tokyo, Bunkyo, Tokyo 113-8656, Japan; 2 Graduate School of Frontier Sciences, The University of Tokyo, Kashiwa, Chiba 277-8561, Japan; murayama@edu.k.u-tokyo.ac.jp; 3 Japan Aerospace Exploration Agency, Mitaka, Tokyo 181-0015, Japan; wada.daichi@jaxa.jp

**Keywords:** fiber optic sensor, fiber Bragg grating, distributed sensing, strain and temperature, dynamic sensing, evaluation

## Abstract

A novel method is introduced in this work for effectively evaluating the performance of the PANDA type polarization-maintaining fiber Bragg grating (PANDA-FBG) distributed dynamic strain and temperature sensing system. Conventionally, the errors during the measurement are unknown or evaluated by using other sensors such as strain gauge and thermocouples. This will make the sensing system complicated and decrease the efficiency since more than one kind of sensor is applied for the same measurand. In this study, we used the approximately constant ratio of primary errors in strain and temperature measurement and realized the self-evaluation of the sensing system, which can significantly enhance the applicability, as well as the reliability in strategy making.

## 1. Introduction

In recent years, fiber optic sensors (OFSs) have been attracting attention in many fields including structural health monitoring (SHM) for their advantages of being small in size, electricity independence in the sensing head, immunity to electromagnetic interference [1], multi-parameter (strain, temperature, etc.) sensitivity, as well as the capability of distributed measurement [2]. With all the advantages mentioned above, the fiber Bragg grating (FBG) sensor, a kind of OFS, is being widely developed for the SHM of various structures such as aircraft and civil infrastructure [3,4]. However, in practical applications, large variations of environmental temperature could bring adverse effects to the strain measurement. Thus, the compensation of thermal effect is one of the key issues to be addressed. Other than employing a stress-free reference FBG for temperature compensation that is used conventionally, multiple parameter FBG sensors are attracting a lot of attention. Over the past few decades, various FBGs have been used for the discrimination of strain and temperature, such as PANDA type polarization-maintaining fiber Bragg grating (PANDA-FBG) [5], two superimposed FBGs [6], superstructure FBG [7], etched core FBG [8], and tilted FBG [9]. Among the reported solutions, PANDA-FBG has shown good potential in distributed measurement. In 2011, Wada et al. applied a 100 mm PANDA-FBG to an optical frequency domain reflectometry (OFDR), and succeeded in simultaneous distributed measurement of strain and temperature statically [10]. Later, the discrimination of dynamic strain and temperature distribution was demonstrated by Zhu et al. using 300 mm high birefringence PANDA-FBG [11].

Practically, unexpected local failures, such as decay of FBG and physical damage, may occur to the installed sensors, leading to abnormal errors. In order to distinguish them, conventionally, people install strain gauges or thermocouples at some locations as a reference to estimate the level of measurement errors during the operation [12,13,14]. However, the use of reference sensors will make the sensing system more complicated, especially for distributed sensing. In this study, we use the properties of PANDA-FBG’s simultaneous strain and temperature sensing system and realize the “self-evaluation”, referring to the error estimation without using other reference sensors. To our best knowledge, this is the first time that by only using the optical fiber itself the dynamic distributed error levels are available to be estimated. The employment of this technique can make further improvement of the sensing system’s applicability and reliability.

## 2. Dynamic Simultaneous Strain and Temperature Measurement

For the measurement of strain and temperature simultaneously, an FBG inscribed in high birefringence PANDA fiber ($8.77\times 10^{-4}$, custom made, Fujikura, Co., Ltd, Tokyo, Japan) is employed. Due to the birefringence, two distinct Bragg wavelengths can be observed at orthogonal polarization states. The values can be calculated as

(1) $$\lambda_{s,f}=2n_{s,f}\Lambda ,$$

where $\lambda_{s,f}$ is the Bragg wavelength of slow or fast mode, $n_{s,f}$ is the effective refractive index of slow or fast mode, and Λ is the period of FBG, respectively. Each Bragg wavelength has different responses to external variation of strain and temperature, which is expressed as

(2) $$\left(\begin{array}{c} \Delta \lambda_{f}\\ \Delta \lambda_{s} \end{array}\right)=\left(\begin{array}{cc} K_{\varepsilon f} & K_{Tf}\\ K_{\varepsilon s} & K_{Ts} \end{array}\right)\left(\begin{array}{c} \Delta \varepsilon \\ \Delta T \end{array}\right)=K\left(\begin{array}{c} \Delta \varepsilon \\ \Delta T \end{array}\right),$$

where $\Delta \lambda_{f}$ and $\Delta \lambda_{s}$ are the wavelength shifts of fast and slow modes, and Δε and ΔT are the strain and temperature variations. *K* represents the coefficient of strain or temperature sensitivities, and the subscripts f,s,ε and *T* correspond to the fast mode, slow mode, strain and temperature, respectively. Based on this linear assumption, we can determine the strain and temperature changes at the same time by measuring the two Bragg wavelength shifts, which are expressed as

(3) $$\left(\begin{array}{c} \Delta \varepsilon \\ \Delta T \end{array}\right)=K^{-1}\left(\begin{array}{c} \Delta \lambda_{f}\\ \Delta \lambda_{s} \end{array}\right).$$

**Figure 1:**
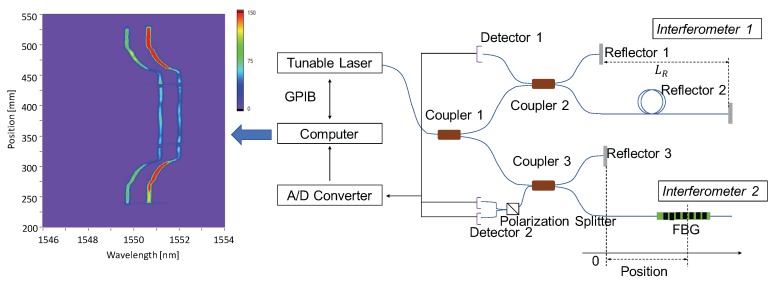
The schematic of the sensing system used in this study. All the couplers and optical fibers are polarization maintaining. The spectrogram on the left is the measurement of a 300 mm PANDA fiber Bragg grating used for system test [19].

In order to obtain the Bragg wavelength distributions of fast and slow modes, respectively, a polarization maintaining OFDR is employed. As shown in Figure 1, the system generally includes one tunable laser source (TLS, GQ5510P, Anritsu Co., Atsugi, Japan [15]) and two interferometers. Interferometer 1 generates an cosine clock signal, $I_{D1}$, which is expressed as

(4) $$I_{D1}=cos\left(2nL_{R}k\right),$$

where n=1.46 is the refractive index, $L_{R}=2\;m$ is the relative distance between Reflector 1 and 2, and *k* is the wavenumber, respectively [16]. It triggers the signal acquisition of Interferometer 2 with a constant wavenumber interval ($\Delta k=\pi /(nL_{R})$). This eliminates the wavelength detection errors caused by the nonlinearity of TLS wavelength sweeping (sinusoidal in this study). When $45^{\circ }$ linear polarized light is injected through Coupler 1, the same amount of reflected light of slow and fast modes are discriminated by the Polarization Splitter before Detector 2. For signal processing, we apply short-time Fourier transform (STFT) to the signal from Detector 2. In this case, the time and frequency in the usual STFT correspond to the wavenumber and frequency, respectively. We set the sweep range of TLS as 1537–1568 nm, and the sweep period as 1/1250s. Considering the time consumed by data transfer, the actual maximum sampling rate is 800 S/s. Additionally, by applying 400pm sliding window to STFT, the spatial resolution, referring to the 10–90% rise distance of a transition of measurand [17], is as high as 1 mm [10,11]. Based on the Nyquist–Shannon sampling theorem, the maximum measurement distances should be $L_{R}/2=1\;m$ [18].

**Figure 2:**
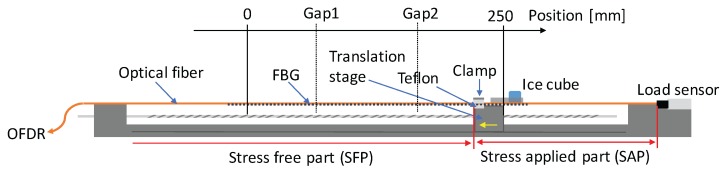
The schematic of test equipment. The right end of fiber under test (FUT) is fixed onto a load sensor, while the middle is clamped onto the translation stage on a trail. Two pieces of Teflon are inserted between the clamp and FUT, and a pure ice cube is put on the shelf above. The yellow arrow on the translation stage indicates the moving direction of it.

To demonstrate the actual dynamic performance of the system, a set of equipment is used, as shown in Figure 2 [11]. Since we applied constant normal force at the clamping surface, when the translation stage is moving, constant strain can be applied to the stress applied part (SAP) by kinetic friction, of which the exact value (*P*) is monitored by the load sensor. Then, the applied strain can be calculated as

(5) $$\varepsilon =\frac{4P}{\pi Ed^{2}},$$

where E=59.2GPa is the Young’s modulus of fiber under test (FUT), and d=142μm is the diameter of FUT, respectively. Meanwhile, the temperature distribution is applied by a pure ice cube. Since the FUT will be covered by the continuously melted water on the bottom of ice cube, 0 ${}^{\circ }C$ temperature can be stably applied.

**Figure 3:**
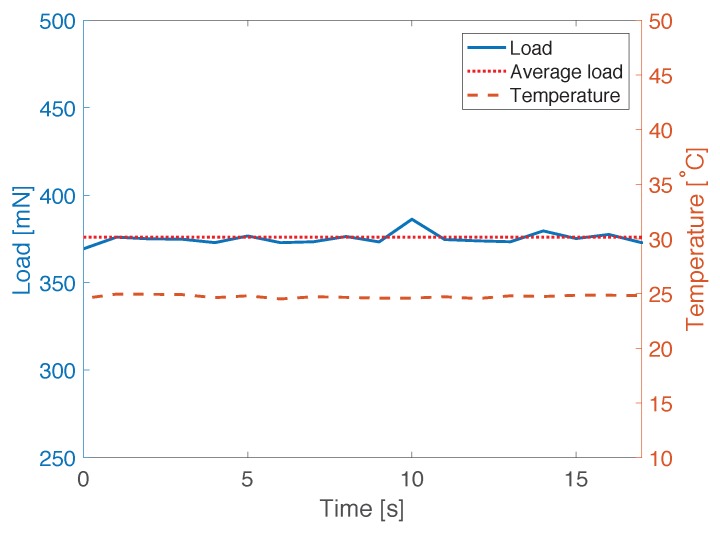
Monitored load and temperature.

In this study, the length of PANDA-FBG is 300mm, and the Bragg wavelength of slow mode is 1550nm. In addition, there are two ∼5 mm gaps at the positions of 60 mm and 160 mm, where no FBG was inscribed. They can generate similar condition as the decay of FBG, which will cause abnormal errors at corresponding positions. In the demonstration, we installed the FUT to the test equipment and moved the stage from the left hand side to the right hand side at the speed of ∼8.2 mm/s. The time when the left edge of the ice cube was at position 200 mm was set to be 0 s. At the same time, the Bragg wavelengths were measured by the OFDR system at 800S/s. Then, the distributions of strain and temperature were calculated by Equation (3). The values of the **K** matrix were calibrated to be $K_{\varepsilon f}=1.22\;\mathrm{pm}/\mu \varepsilon$, $K_{\varepsilon s}=1.23\;\mathrm{pm}/\mu \varepsilon$, $K_{Tf}=11.8\;\mathrm{pm}{/}^{\circ }C$, and $K_{Ts}=11.1\;\mathrm{pm}{/}^{\circ }C$. During the test, as shown in Figure 3, the room temperature was monitored to be $25\pm 0.5{\;}^{\circ }C$ by thermocouple, and the measured friction by load sensor was 375.0±3.0mN from 0 s to 17 s. According to Equation (5), the corresponding applied strain was calculated to be 400.0±3.2με.

**Figure 4:**
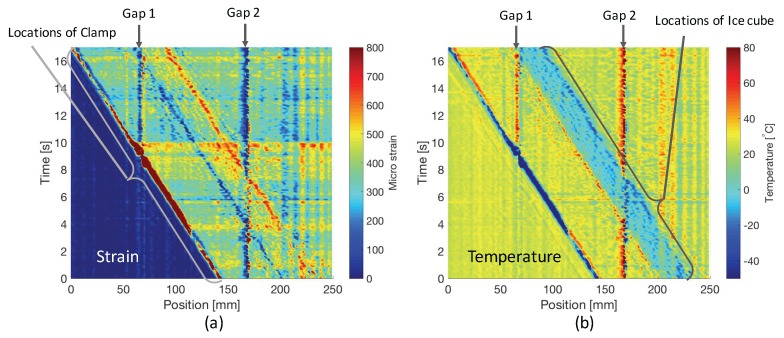
(**a**) measured variation of strain distribution the strain variation; (**b**) measured variation of temperature distribution.

**Figure 5:**
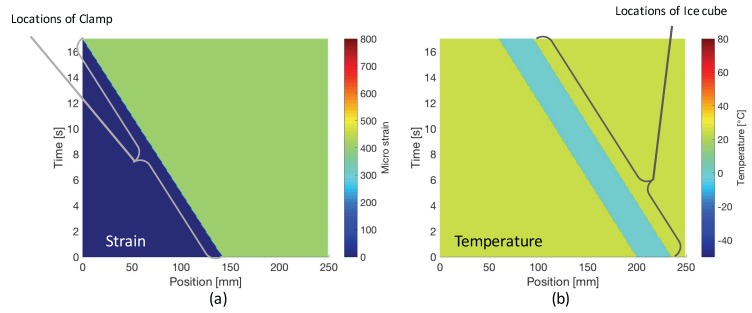
(**a**) simulated variation of strain distribution; (**b**) simulated variation of temperature distribution.

As shown in Figure 4, the variations of strain and temperature distribution over time have been successfully obtained simultaneously. The movements of the clamp, ice cube, as well as two constant gaps can be clearly observed. Meanwhile, by using the monitored data from reference sensors (load sensor and thermocouple), the simulated variations are shown in Figure 5.

## 3. Conventional Evaluation

In practical applications, various local failures might occur to the sensors including the thermal decay of FBG, physical damage, etc. The decay of FBG might significantly decrease the reflectivity, leading to bad signal noise ratio, and the physical damage might block the light path or cause broadband reflection, respectively, at corresponding positions. Both of them can cause large measurement errors that will influence the reliability of the sensing system. Thus, the evaluation of sensors during the measurement is necessary.

**Figure 6:**
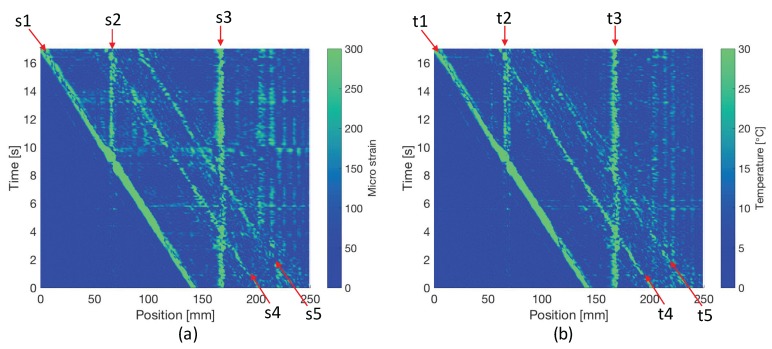
(**a**) estimated error levels for strain measurement. The marks from s1 to s5 represent five groups of abnormal errors in strain measurement; (**b**) estimated error levels for temperature measurement. The marks from t1 to t5 represent five groups of abnormal errors in temperature measurement.

Conventionally, we evaluate the sensing performance by comparing the values obtained by FBG with the ones obtained by reference sensors. In this study, the levels of error are defined as the absolute value of the difference between measurement values and simulated reference ones, which is expressed as

(6) $$\begin{array}{c} Err_{c,\varepsilon }\left(p,t\right)=\left|\varepsilon_{m}\left(p,t\right)-\varepsilon_{r}\left(p,t\right)\right|, \end{array}$$

(7) $$\begin{array}{c} Err_{c,T}\left(p,t\right)=\left|T_{m}\left(p,t\right)-T_{r}\left(p,t\right)\right|, \end{array}$$

where $Err_{c,\varepsilon }\left(p,t\right)$, $Err_{c,T}\left(p,t\right)$ are conventionally estimated error levels of strain and temperature at position *p* and time *t*. $\varepsilon_{m}\left(p,t\right)$, $\varepsilon_{r}\left(p,t\right)$, $T_{m}\left(p,t\right)$, and $T_{r}\left(p,t\right)$ are measured strain, simulated reference strain, measured temperature, and simulated reference temperature, respectively. By calculating at every position and time, the results of the error levels are shown in Figure 6. In this study, if the detected error is larger than 10 times the accuracy (∼16.7 με and ∼1.9 ${}^{\circ }C$ [19]), we can regard it as abnormal. Among the abnormal errors, s1, s4, s5, t1, t4, and t5 are caused by the phase differences of fast and slow modes at the positions where steep changes of strain or temperature occur. Meanwhile, s2, s3, t2 and t3 are abnormal errors caused by the two constant gaps (local failure, or dead zones) on the FBG.

## 4. Self-Evaluation

Although in the last section the reference sensors work well for retrieving the abnormal errors during the measurement, they will become less applicable in practical cases, especially when the FBG sensor is bonded or embedded. Thus, the development of a fast and reliable evaluation function of the system is necessary. In this section, a novel self-evaluation method for distributed PANDA-FBG sensor is introduced.

In simultaneous measurement, various errors may come from the Bragg wavelength detection, calibration of **K** matrix, local failure (dead zone) of sensor, etc. By using Equation (3), their relations can be expressed as

(8) $$\left(\begin{array}{c} \Delta \varepsilon +\delta \varepsilon \\ \Delta T+\delta T \end{array}\right)=\left(K^{-1}+\delta K_{\mathrm{inv}}\right)\left(\begin{array}{c} \Delta \lambda_{f}+\delta \lambda_{f}\\ \Delta \lambda_{s}+\delta \lambda_{s} \end{array}\right),$$

(9) $$\left(\begin{array}{c} \delta \varepsilon \\ \delta T \end{array}\right)=K^{-1}\left(\begin{array}{c} \delta \lambda_{f}\\ \delta \lambda_{s} \end{array}\right)+\delta K_{\mathrm{inv}}K\left(\begin{array}{c} \Delta \varepsilon \\ \Delta T \end{array}\right)+\delta K_{\mathrm{inv}}\left(\begin{array}{c} \delta \lambda_{f}\\ \delta \lambda_{s} \end{array}\right),$$

where δε is the error of strain, δT is the error of temperature, $\delta K_{\mathrm{inv}}$ is the error of inversed matrix **K**, $\delta \lambda_{f}$ is the error of wavelength shift of fast mode and $\delta \lambda_{s}$ is the error of wavelength shift of slow mode [19]. Among the terms on the right-hand side of Equation (9), the first one is dominating the value of errors. Considering the relations of $K_{\varepsilon f}\approx K_{\varepsilon s}$ and $K_{Tf}\approx K_{Ts}$, the first term can be derived into

(10) $$Err_{main}=K^{-1}\left(\begin{array}{c} \delta \lambda_{f}\\ \delta \lambda_{s} \end{array}\right)\approx \frac{\delta \lambda_{f}-\delta \lambda_{s}}{K_{\varepsilon f}K_{Ts}-K_{Tf}K_{\varepsilon s}}\left(\begin{array}{c} K_{Ts}\\ -K_{\varepsilon s} \end{array}\right).$$

**Figure 7:**
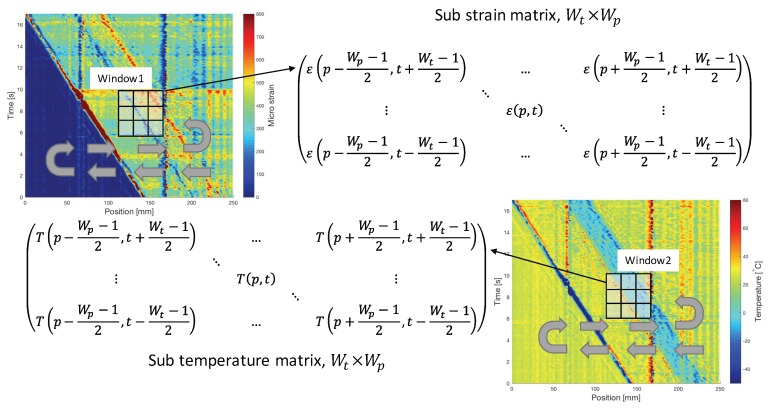
The definition of sliding windows. Two windows are sliding in the direction of the arrows: from 250 mm to 0 mm and from 0 s to 17 s. (p,t) is the position of window center, $W_{p}$ is the width of window at position domain, and $W_{t}$ is the width of window at time domain. Both $W_{p}$ and $W_{t}$ should be odd numbers larger than 1.

From the derived relation, we can see that errors of strain and temperature always have an approximately constant ratio of $-K_{Ts}/K_{\varepsilon s}$, about −9, which can also be observed in Figure 4 and Figure 6. Thus, the principle of the self-evaluation is to find the positions and times where and when similar opposite profile of errors in strain and temperature measurement are detected, and then retrieve their absolute values, respectively. To realize it, an algorithm based on 2D correlation which is typically used to detect similarities between two 2D images [20] was developed. As shown in Figure 7, we synchronously slide two windows of the same size ($W_{p}\times W_{t}$) in the 2D graphs formed with position and time domain information of the measurements to select sub strain and temperature matrices.

**Figure 8:**
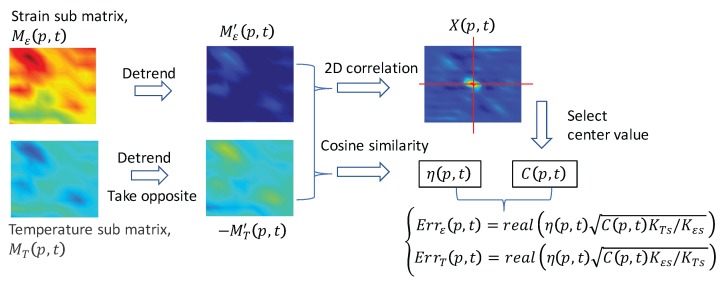
Flow chart of the self-evaluation process.

Figure 8 shows the process of self-evaluation. The selected sub matrices $M_{\varepsilon }\left(p,t\right)$ and $M_{T}\left(p,t\right)$ are firstly detrended by using the best plane fit. Then, the fast 2D correlation between the detrended matrices ($M_{\varepsilon }^{\prime }\left(p,t\right)$ and $M_{T}^{\prime }\left(p,t\right)$) is calculated as

(11) $$X\left(p,t\right)=FFT_{2D}^{-1}\left\{FFT_{2D}\left\{M_{\varepsilon }^{\prime }\left(p,t\right)\right\}FFT_{2D}{\left\{-M_{T}^{\prime }\left(p,t\right)\right\}}^{*}\right\},$$

where $FFT_{2D}$ and $FFT_{2D}^{-1}$ indicate the operation of 2D fast Fourier transform and its inverse, respectively. Meanwhile, the cosine similarity of the detrended matrices is calculated as

(12) $$\eta \left(p,t\right)=\frac{V_{\varepsilon }\cdot V_{T}}{∥V_{\varepsilon }{∥}_{2}{∥V_{T}∥}_{2}},$$

where $V_{\varepsilon }$ and $V_{T}$ are one-dimensional column vectors reshaped from matrices $M_{\varepsilon }^{\prime }\left(p,t\right)$ and $-M_{T}^{\prime }\left(p,t\right)$, respectively [21,22]. By considering the constant ratio of strain and temperature errors at the same position and time, the error levels can be retrieved by

(13) $$Err_{s,\varepsilon }\left(p,t\right)=real\left(\eta \left(p,t\right)\sqrt{C\left(p,t\right)K_{Ts}/K_{\varepsilon s}}\right),$$

(14) $$Err_{s,T}\left(p,t\right)=real\left(\eta \left(p,t\right)\sqrt{C\left(p,t\right)K_{\varepsilon s}/K_{Ts}}\right),$$

where $Err_{s,\varepsilon }\left(p,t\right)$, $Err_{s,T}\left(p,t\right)$ and C(p,t) are self-evaluated error level of strain, temperature and the center element of matrix X(p,t), respectively. It is worth noticing that we have taken the opposite value of $M_{T}^{\prime }\left(p,t\right)$ in Equation (11); thus, only the real parts in Equations (13) and (14) are taken into account.

## 5. Results

**Figure 9:**
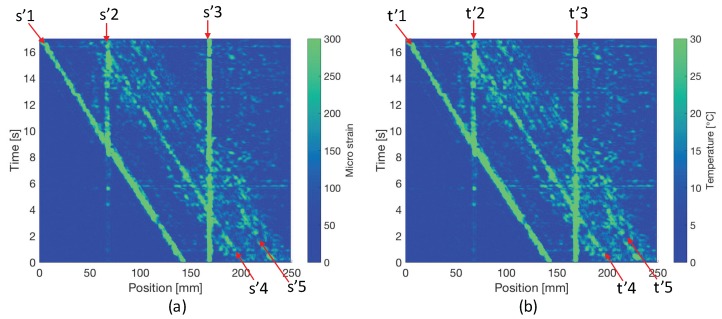
(**a**) self-evaluated error levels for strain measurement; (**b**) self-evaluated error levels for temperature measurement.

In this study, we use two 3×3 windows, and conduct the self-evaluation for the complete measurement, of which the results are shown in Figure 9. In total, 10 groups of abnormal errors are successfully observed at the same locations as in Figure 6. Among them, s’2, s’3, t’2 and t’3 are abnormal errors caused by the Gap1 and Gap2 as shown in Figure 2, and the rest are caused by the phase differences between fast and slow mode at the positions where steep variations of strain or temperature occur.

## 6. Discussion

In order to give quantitative comparisons of self-evaluated results and conventional estimated results using reference sensors, the 2D Pearson’s correlation coefficients and cosine similarities of Figure 6 and Figure 9 are calculated, as given in Table 1. Statistically, in the comparisons of both strain and temperature, strong positive linear relations [23] and high similarities are shown with the values of correlation coefficient and cosine similarity, respectively.

Considering the measurement results shown in Figure 4, Gap1 and Gap2 give good simulation of local failures in the sensing FBG, which will introduce confusing measured signals and disturb our strategy making. However, by taking self-evaluated error levels as a reference, the large abnormal errors at ∼60 mm and ∼160 mm through the whole test obviously point out that there are two dead zones at corresponding positions. Additionally, the self-evaluation shows that the error levels at the front edge of clamp and both edges of ice cube are very high. Thus, those measured values should not be directly used for detecting stress concentration, damage, etc. In general, this technique provides us with very direct references for the post data analysis and strategy making in the dynamic strain and temperature distribution measurement.

It is worth mentioning that, by using 3×3 windows, slightly lower spatial resolution but no obvious delay in time are observed in the self-evaluation results. However, if larger windows are applied, the spatial resolution will become even lower. Meanwhile, the significantly increased calculation will delay the self-evaluation and sampling rate that might make the self-evaluation not suitable for real time application.

Furthermore, the principle of self-evaluation is not limited to the PANDA-FGB sensor, but also shows good potential in other simultaneous measurement techniques, such as Brillouin scattering, Rayleigh scattering, etc [24,25]. Thus, the expansion of the application of self-evaluation is one of our future works.

## 7. Conclusions

In our study on the PANDA-FBG sensing system for simultaneous dynamic strain and temperature distribution measurements, we introduce a self-evaluation method by using the characteristics of error generation during the sensing process. This novel method gives precise detection of the position and time of abnormal errors without the use of extra reference sensors, which significantly simplified the sensing system and brought further enhancement of its reliability and applicability. In addition, the principle of self-evaluation might be expanded to other simultaneous sensing techniques in future works.

## Figures and Tables

**Table 1:** The comparison of self-evaluation and conventional estimation.

Name	Strain	Temperature
Correlation coefficient	0.61	0.63
Cosine similarity	0.71	0.71
